# Effectiveness of percutaneous flexor tenotomies for the management and prevention of recurrence of diabetic toe ulcers: a systematic review

**DOI:** 10.1186/s13047-016-0159-0

**Published:** 2016-07-29

**Authors:** Jennifer E. Scott, Gordon J. Hendry, John Locke

**Affiliations:** School of Health and Life Sciences / Institute for Applied Health Research, Glasgow Caledonian University, Cowcaddens Road, Glasgow, G4 0BA UK

**Keywords:** Flexor tenotomy, Toe ulcer, Diabetes, Ulceration, Plantar pressure, Toe deformity

## Abstract

**Background:**

Diabetic toe ulcers are a potentially devastating complication of diabetes. In recent years, the percutaneous flexor tenotomy procedure for the correction of flexible claw and hammer-toe contraction deformities has been proposed as a safe and effective technique for facilitating the healing of toe-deformity related diabetic ulcers. The aim of this review is to critically appraise the evidence for the effectiveness of this surgical procedure in achieving ulcer healing, prevention of re-ulceration, and to summarise the rate of post-operative complications.

**Method:**

A search of medical databases, was performed to locate relevant literature. Titles were screened prior to abstract and full text review to identify articles relevant to the research question. Search terms included truncations of “tenotomy”, “toe”, “hallux”, “digit”, “diabetes” and “ulcer”. Peer reviewed primary research study designs specified as suitable for systematic reviews by the Centre for Reviews and Dissemination were included. Studies were excluded if they used a concurrent secondary procedure or included non-diabetic patients without reporting outcomes separately. Included studies were appraised for quality using the Methodological Index for Non-Randomised Studies tool. Levels of evidence were subsequently assigned to each outcome of interest (healing rate and prevention of re-ulceration).

**Results:**

From a total search yield of 42 articles, 5 eligible studies (all case series designs) were identified for inclusion. Included studies were of low-to-moderate methodological quality when assessed using the MINORS tool. A total of 250 flexor tenotomy procedures were performed in a total of 163 patients. Included studies generally reported good healing rates (92–100 % within 2 months) post-op follow-up), relatively few recurrences (0–18 % at 22 months median post-op follow-up), and low incidences of infection or new deformity. Transfer ulcers developing on adjacent areas as a result of shifted pressure were reported by several authors.

The validity of these results is undermined by methodological limitations inherent to case series designs such as a lack of control groups, non-randomised designs, as well as inconsistent reporting of post-intervention follow-up periods. There was level 4 evidence for the flexor tenotomy procedure in facilitating ulcer healing and preventing re-ulceration.

**Conclusion:**

More definitive research evidence is needed in this area to determine whether or not the flexor tenotomy is a safe and effective treatment option for people with, or at risk of developing diabetic toe ulcers. Whilst the available literature reports that the procedure may be associated with high healing rates, relatively low recurrence rates and low incidences of post-op complications, methodological limitations restrict the value of these findings.

**Electronic supplementary material:**

The online version of this article (doi:10.1186/s13047-016-0159-0) contains supplementary material, which is available to authorized users.

## Background

Foot ulceration can be a devastating complication of diabetes. The dorsum, apices and plantar aspects of the toes are particularly vulnerable locations for diabetic foot ulcers, with studies showing ulcers occurring on toes 1–5 accounting for between 43 and 55.5 % of all foot ulcers [1, 2]. Whilst digital ulcers tend to be smaller and heal faster than forefoot, mid-foot or heel ulcers [3], they may be an important prognostic indicator given that they may precede up to 63.9 % of diabetic limb amputations [4], the corollary being that timely resolution and prevention of toe ulceration is crucial to avoid poor long-term outcomes.

Toe deformities such as ‘hammer’ and ‘claw’ toes have been associated with the development of diabetic foot ulceration, particularly in the presence of complicating factors such as neuropathy and peripheral vascular disease [5]. During weight-bearing or gait, insensate and deformed toes may be subject to increased pressures and shear stresses, which can result in callous formation, tissue trauma, and ultimately ulceration [6].

Off-loading pressure from ulcer sites is considered to be an important treatment goal for promoting healing and preventing the ulcer from recurring [7] Historically the conventional approach to pressure offloading has been conservative through debridement of superficial skin lesions (corns, callus), deflective padding, insoles, and/or therapeutic footwear [6, 8, 9]. However robust evidence of the efficacy of such interventions is lacking and what does exist is confounded by poor patient adherence. Indeed, patient adherence to wearing removable offloading devices such as pressure relieving footwear has been reported as being particularly problematic [10].

Researchers have suggested that certain surgical interventions can reduce the risk of foot ulcer recurrence in patients with diabetes and peripheral neuropathy [11]. Minimally-invasive surgical procedures may reduce infection and healing rates [12]. The flexor tenotomy procedure is advocated for flexible toe deformities [13] and can be performed on both the hallux and lesser toes. During this operation, the flexor digitorum/hallucis longus tendon is transected under local anaesthetic through a single plantar incision [14], with some surgeons also choosing to release the flexor digitorum/hallucis brevis tendon [15]. The therapeutic aim is to release the flexor digitorum brevis/longus tendon contraction, allowing the toe to adopt a straighter position in order to alleviate the focal pressure on areas of ulceration, in particular the toe apices. The procedure is generally conducted on an outpatient basis.

A systematic review [8] published in 2009 sought to establish whether or not the flexor tenotomy procedure was safe and effective for the treatment of toe ulcers in diabetic patients. This review concerned neuropathic DFUs and was limited to the evaluation of two case-series studies [16, 17] which were deemed to be of poor methodological design, but which indicated that the procedure may result in healing of digital ulcers with a low incidence of complications [8]. Since this review several new studies on this procedure have been published, widening the body of evidence on this procedure, and necessitating an updated review of the available literature. Accordingly, the aim of this review is to critically evaluate the available literature to establish if there is sufficient evidence to demonstrate that the flexor tenotomy for diabetic ulcers on the apices of the hallux and lesser toes is 1) effective in achieving ulcer healing, 2) effective in preventing recurrence of ulcers, and 3) to summarise the rates of post-operative complications.

## Methods

### Search strategy

During 1st–30th September 2015, Pubmed and EBSCO Host (incorporating AMed, CINAHL, Health Source and MEDLINE) databases, as well as the Cochrane Library and World Health Organisation International Clinical Trials Registry were searched by the first author for relevant literature. The reference lists of relevant articles were also searched by hand.

The search terms, truncation and Boolean operators used to search the databases are outlined in Table 1. Given that the flexor tenotomy is an emerging surgical approach, no filters were applied (i.e. all fields were searched) in order to ensure the widest possible return of results. The earliest reference found for the use of the procedure in toe deformities in any patient population dated to 1975 [18]. All literature from this year onwards was included in the search.

**Table 1:** Literature Search Terms

Terms	Rationale
1. Tenotom* AND	1. No alternative synonym for tenotomy was located.
2. (toe* OR hallux OR digit*) AND	2. Tenotomies are performed on many joints e.g. knee and shoulder. Specific anatomical location is required.
3. (diabet* OR ulcer*)	3. ‘Diabetes’ and ‘Ulcer’ keywords ensure relevance to research population.

*representes the use of truncation

### Inclusion and exclusion criteria

**Fig. 1:**
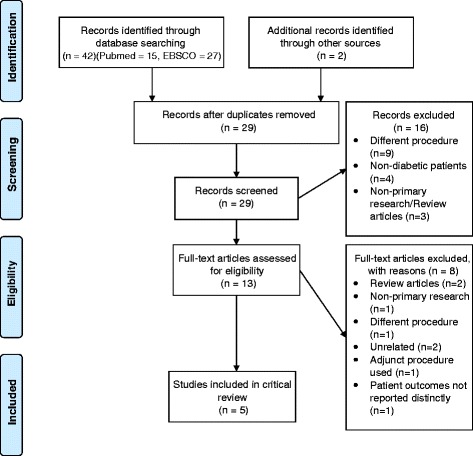
PRISMA Diagram (Adapted from Moher et al. [30])

Forty-two titles and abstracts were screened by the first author to eliminate duplicates and obviously irrelevant articles. The first author then accessed the full texts of the remaining 13 studies and reviewed them against the inclusion and exclusion criteria to identify articles relevant to the research question. The process of selecting the included papers is summarised in Fig. 1, with the full list of papers excluded included in an additional file [See Additional file 1].

Peer reviewed primary research study designs specified as suitable for systematic reviews by the Centre for Reviews and Dissemination (CRD) [19] were included. These comprise randomised controlled trials, quasi-experimental studies and observational studies. Studies reporting outcomes for the hallux and/or lesser toes were included. As Diabetic Foot Ulcers (DFUs) may be multifactorial, diabetic patient groups with or without neuropathy or peripheral arterial disease were included.

Studies were excluded if they 1) used a concurrent secondary procedure (e.g. osteotomy), 2) were study designs not specified in CRD evidence hierarchy [19] (e.g. reviews, meta-analysis, single-case studies) or 3) Included non-diabetic patients without reporting outcomes separately.

### Data extraction

Data extraction was performed by the first author, and all queries were discussed and resolved by the supervisory team in regular meetings. Data concerning sample size, participant age, gender, diabetes and ulcer characteristics, surgical intervention and surgical outcomes were extracted from the studies directly into a Microsoft Word table using headings relevant to the research question (Table 2, Table 3).

**Table 2:** Literature Review – Study Characteristics

	Kearney et al. [23]	Laborde [17]	Rasmussen et al. [15]	Tamir et al. [24]	Van Netten et al. [25]
Patients (No.)	48	14	16	55	30
Procedures (No.)	58	24	27	103	38
Age range (Years)	• Mean 68.1 ± 2.3	• 40–81	• 37–91	• 48–89	• 42-93
		• Mean 55	• Mean 62.8	• Mean 65	• Mean 69 ± 12
Gender	• M:11, F:37	• M: 7 F: 11	• Insufficiently reported: prophylactic and ulcerated patients reported together	• Not reported	• M: 17, F:16
Diabetes duration	• Not reported	• Not reported	• Insufficiently reported: prophylactic and ulcerated patients reported together	• Insufficiently reported – patients receiving different interventions reported together	• Not reported
HbA1C	• 7.3 ± 1.4	• Not reported	• Insufficiently reported: prophylactic and ulcerated patients reported together	• Insufficiently reported – patients receiving different interventions reported together	• Not reported
Inclusion criteria	• Diabetes	• Not reported	• Not reported	• Not reported	• Not reported
	• Distal toe ulceration				
Exclusion criteria	• Healed ulcer	• Grade 4/5 ulcers^b^	• Not reported	• ABPI below 0.5/flat pulse volume at ankle	• Not reported
	• Adjunct procedures				
				• Cellulitis	
Pre-selection assessments (% of patients with condition)	• Neuropathy: mono-filament/biothesiometer (100 %)	• Neuropathy: mono-filament (100 %)	• Neuropathy: monofilament/biothesiometer (100 %)	• Neuropathy: Assessment and patient characteristics not reported.	• Neuropathy: monofilament (100 %)
		• Poor vascular status: absent pulses (14 %)			• PAD: absent pulses/Doppler (0 %)
	• PAD: absent pulses (36.2 %)				
			• Poor arterial perfusion: Pulses/ankle blood pressure (18 %)		
				• Vascular status: ABPI (% not reported)	
Ulcer grades –No. (% of ulcers)	• Not reported	• 1^b^ – 11 (46 %)	• 1^a^ – 23 (85 %)	• 0^a^ – 25 (24 %)	• 1^a^ – 20 (53 %)
		• 2^b^ – 5 (21 %)	• 2^a^ – 1 (4 %)	• 1^a^ – 73 (71 %)	• 2^a^ – 0 (0 %)
		• 3^b^ – 8 (33 %)	• 3^a^ – 3 (11 %)	• 2/3^a^ – 5 (5 %)	• 3^a^ – 18 (47 %)
Ulcer duration	• Not reported	• 1 month – 5 years	• Range 1–48 weeks	• Range: 1 – 156 Weeks	• 9 – 525 days
					• Mean: 96 days
		• Average: 10 months	• Median: 15 weeks	• Mean: 33 weeks	
Digit tenotomised	• Not specified – only FDL transected, therefore assumed to be digits 2-5	• Hallux – 14	• Hallux – 15	• Hallux – 16	• Hallux – 12
		• 2 – 7	• 2 – 10	• 2 – 31	• 2 – 15
		• 3 – 2	• 3 – 2	• 3 – 37	• 3 – 11
		• 4 – 0	• 4 – 0	• 4 – 16	• 4 – 0
		• 5 – 1	• 5 – 0	• 5 – 3	• 5 – 0
Incision location	Distal phalanx	Proximal portion of proximal phalanx	1 cm proximal to the web fold	Mid-portion of proximal phalanx	Mid-portion of proximal phalanx
Tendons transected	• FDL – 58 toes	• FDL & FDB –	• FDL & FDB –	• FDL – 87 Toes	• FDL – 26 Toes
		• 10toe	• 12 Toes	• FHL – 16 Toes	• FHL – 12 Toes
		• FHL – 14 Toes	• FHL & FHB – 15 Toes		
Post-op offloading	• Immediate weightbearing	• Full weightbearing	• 2–3 days post-op hosp. immobilization	• Not reported	• 24 h offloading plus pressure bandage
		• Post-op shoes/sandals/extra depth shoe			
	• Rigid soled sandals				
			• Rocker bottom sandals + soft insoles		
Return appointment	Not reported	3–5 days then weekly until healed	1 weeks then as required until healed	1 week then regularly until healed	1 week then regularly
Follow-up period (months)	• Mean: 28	• 20–64	• 2–48	• Minimum: 5	• 11–60
					• Mean: 23 ± 11
				• Interquartile range:16–29	
				• Median: 22	
		• Average: 36	• Median: 31		

KEY: *No*. Number, *ABPI* Ankle Brachial Pressure Index, *PAD* Peripheral Arterial Disease, *FDL* Flexor Digitorum Longus, *FDB* Flexor Digitorum Brevis, *FHL* Flexor Hallucis Longus, *FHB* Flexor Hallucis Brevis, ^a^Ulcer Grades on Texas Scale [26], ^b^Ulcer grades on Wagner’s Scale [26]

NOTE: Data reported in the original studies on non-diabetic patients [17], non-Flexor Tenotomy procedures [24] and prophylactic procedures [15, 25] have been omitted from this table due to irrelevance to the research question

**Table 3:** Literature Review - Results

	Kearney et al. [23]	Laborde [17]	Rasmussen et al. [15]	Tamir et al. [24]	Van Netten et al. [25]
Ulcers Healed (% of ulcers)	98.3 %	100 %	93 %	98 %	92 %
Healing Time	• 40 ± 52 days	• Under 2 months	• 7–224 days	• 98 % wound closure within 4 weeks	• 4–154 days
					• Mean 22 ± 26 days
			• Median: 21 days		
Recurrence (% of ulcers)	• 12 %	• 8 %	• 11 %	• 0 %	• 18 %:
		• 2 hallux ulcers	• 3 ulcers – not specified		• 4 lesser toes, 3 first toes
	• 7 lesser toe ulcers				
Time to recur	13.9 ± 15.2 months	45–48months	Not reported	Not applicable	Not Reported
Infections	2 Ulcers (3 %)	0 %	0 %	1 Patient (2%)	0 %
Complications	• 1 Amputation: ulcer non-healing due to pre-existing osteomyelits	• No infections or new deformities occurred	• 2 transfer lesions (7 % of procedures) developed 5 and 7 months after surgery	• 2 ulcers non-healing due to insufficient offloading/arterial insufficiency	• 3 Amputations: ulcers non-healing due to pre-existing infection to bone
				• 9 Transfer lesions within 8 weeks of surgery (9 % of procedures)	• 8 shifted (transfer) ulcers (21 % of procedures)
				• 3 Plantar skin ruptures	• Dorsiflexion of the Metatarso-phalangeal joint.
				• 1 Pain	

### Quality appraisal and evidence grading

The quality of the selected studies was assessed using the Methodological Index for Non-Randomised Studies (MINORS) tool [20] by two independent reviewers (JS under supervision from JL, and GJH respectively). Discrepancies in scoring were moderated by a third reviewer (JL) and the final scores agreed upon by the reviewer team. The MINORS tool is a validated instrument specifically designed to overcome inherent difficulties in evaluating surgical studies where randomisation, control groups and blinding are challenging to achieve [21]. The MINORS evaluation allowed notable similarities and differences in the selected studies to be highlighted and synthesised [See Additional file 2]. Following the analysis and synthesis of the extracted data, an evidence rating was assigned according to Oxford Centre for Evidence-based Medicine evidence grading system [22].

## Results

### Search yield

Five primary research papers met the criteria for appraisal in this review; Kearney et al. [23], Laborde [17], Rasmussen et al. [15], Tamir et al. [24] and Van Netten et al. [25].

### Participant characteristics

The numbers of non-prophylactic procedures performed in the 5 selected studies totalled 250, ranging from 24 in Laborde [17] to 103 in Tamir et al. [24]. The total patient numbers across the studies totalled 163 (Range 14–55) as several patients had multiple tenotomies. All authors obtained patient, procedure and outcome data from retrospective review of medical charts and reported participant characteristics using descriptive statistics to varying levels of detail (Table 2, Table 3). Laborde [17] and van Netten et al. [25] provided relatively comprehensive tables on individual patient characteristics, outcomes and complications, facilitating a more detailed evaluation of their findings.

### Ulcer characteristics

All studies, with the exception of Kearney et al. [23], obtained baseline ulcer characteristics using the University of Texas (UT) or Wagner ulcer classification systems [26]. Tamir et al. [24] performed the majority of procedures on low-complexity ulcers, with only 5 % of lesions classified as UT Grade 2 or 3 (penetrating to tendon/capsule or bone/joint). UT Grade 2 and 3 ulcers accounted for 54, 15 and 47 % of procedures in Laborde [17], Rasmussen et al. [15] and van Netten et al. [25] respectively. All ulcers were located on the distal apex of the toe, and all authors operated on both the hallux and lesser toes, with the exception of Kearney et al. [23] who reported no hallux procedures.

### Intervention

All surgeons performed the procedure using a single percutaneous incision, with variations in the tendons cut and the location of the incision shown in Table 2. All authors chose to release flexor hallucis/digitorum Longus (FHL/FDL), however Laborde [17] and Rasmussen et al. [15] also chose to transect flexor hallucis/digitorum Brevis (FHB/FDB).

### Post-operative care

When reported, post-operative measures varied. Several authors [15, 17, 23] reported prescribing offloading footwear after surgery. Rasmussen et al. [15] appear to have employed a particularly rigorous post-operative regime, mandating hospital admission, 2–3 days of immobilisation and prophylactic antibiotics.

### Healing, recurrence and infection

All studies provided descriptive statistics reporting high healing rates (92–100 % at up to 227 days) and few recurrences (0–18 % at 22 months median follow-up) (Table 3). Van Netten et al. [25] found a statistically significant association between UT ulcer classification and healing time (*p* = 0.042), with more complex ulcers taking longer to heal. In this study, healing was not achieved in three patients, all of whom had UT grade 3B ulcers (infected wound, penetrating to bone) and subsequently underwent digital amputation. Van Netten et al. [25] noted that all seven recurrences in their study occurred in toes with infection penetrating to bone (UT Grade 3B ulcers), while Laborde [17] noted that both recurrences in his study occurred following ulcers probing to tendon (Wagner’s Grade 2 ulcers) [26].

### Follow-up

All studies reported a mean or median follow-up between 22 and 36 months, however the minimum follow-up periods reported in Rasmussen et al. [15] and Tamir et al. [24] were two and five months respectively. Only Laborde [17] provided any information on the follow-up protocol, reporting that 12 patients were assessed in person and 6 by telephone.

### Complications

The studies report relatively low incidences of infection or new deformity (Table 3). Transfer ulcers (ulcers developing on adjacent areas as a result of shifted pressure) were reported by several authors. Rasmussen et al. [15] recorded two transfer ulcers and Tamir et al. [24] reported nine. Of the 38 tenotomies performed by Van Netten et al. [25], 8 were performed on toes that had developed transfer ulcers from an adjacent tenotomised digit. It could be argued that the 8 tenotomies performed on toes following a prior procedure were not in fact classic diabetic foot ulcers, and were instead sequelae of surgery and should have been discounted from the cases reported in their article. The authors report that these eight ‘shifted’ ulcers healed and did not recur, nor result in any further transfer lesions.

### Quality and evidence grading

The studies reviewed scored between 5 and 8 out of an available score of 16, indicating low methodological quality [see Additional file 2]. There is level 4 evidence to support the statement that flexor tenotomy is effective for the healing of and the prevention of digital ulcers in diabetes.

## Discussion

Healing diabetic toe ulcers can be a challenging and protracted process with high recurrence rates, negatively affecting the patients’ quality of life and potentially leading to amputation. It is therefore highly important to establish effective interventions to reduce these negative consequences. Overall, the studies included in this review describe promising results following flexor tenotomy with regard to ulcer healing and recurrence rates: an average of 96.3 % of ulcers healed, and an average of 9.8 % recurred. It would thus appear that the Flexor Tenotomy procedure may be an effective intervention for healing and reducing recurrence of diabetic toe ulcers. The procedure also appears to have a low incidence of complications, with the exception of transfer ulceration, which is not discussed in depth in the studies.

While reported outcomes are encouraging, several aspects of the studies deserve further discussion. All five publications described in this review are retrospective case series, and are therefore vulnerable to bias. The absence of randomisation to a control group means that any changes observed are the result of an uncontrolled pre-post analysis instead of a comparative analysis with control subjects who receive an alternative treatment such as current standard conservative care. Furthermore, the retrospective nature of the data collection introduces the potential for selection bias; indeed, none of the studies provide clear outlines of their sampling processes. The small sample size of the studies poses a further difficulty in generalising the findings.

Differences in surgical technique, specifically tendon choice for resection, may introduce significant heterogeneity between studies, impairing comparability and assessment of the merits of each technique. Rasmussen et al. [15] prefer to sever both FDL/FHL and FDB/FHB tendons to provide maximum relaxation of the flexion deformity, whereas Van Netten et al. [25] imply that severing both tendons is not without complications. They report that inadvertently severing FHB in one of their subjects may have accounted for over-extension of one toe that lead to a dorsal transfer ulcer forming. Laborde [17] attributes the recurrence of three first toe ulcers in his study to a failure to transect FHB.

Studies have shown that, certain types of post-operative footwear can reduce plantar pressures in the forefoot and hallux and thus could impact upon ulcer healing/recurrence rates [27], therefore the impact of post-operative management should have been discussed in the studies. While most of the studies employed some form of footwear offloading, protocols are only briefly discussed and are not sufficient to inform robust comparison. It is probable that the provision of offloading footwear itself may have had a confounding influence on outcomes.

The low incidence of adverse events and ulcer recurrence must be interpreted cautiously due to deficiencies in reporting, most notably due to the lack of consistent and rigid patient follow-up protocols. Indeed it is recognised that spontaneous reporting of adverse effects from medical record review is not a sufficiently rigorous method [28]. Given that ulcer recurrence and post-op complications require time to manifest, the follow-up period must be sufficient, standardised across studies, and include all study participants to ensure accurate reporting. No minimum recommended follow-up periods are unequivocally accepted in the wider literature on digital surgery and diabetic ulceration. However, a period of one year has been deemed a reasonable follow-up period based on SIGN [29] guidelines which recommend that patients at high-risk of ulceration are assessed annually by a specialist podiatrist. In the case of the minimum follow up times reported in Rassmussen et al. [15] and Tamir et al. [24] it could be argued that ulcer recurrence and complications were not adequately monitored.

The means of conducting follow up is also vague across the studies. Laborde [17] describe a follow-up protocol using both telephone and in-person follow-up. However, it is unclear as to whether or not telephone-based follow-ups are safe and effective as these would likely require a certain level of health literacy amongst patients. In the absence of any reporting by the other authors, the validity of their reported post-operative recurrence and complication rates cannot be determined.

## Conclusion

Favourable short-term results are reported across the studies included in this review, suggesting that the flexor tenotomy may be an effective intervention for achieving toe ulcer healing and preventing ulcer recurrence. However these results should be interpreted with caution due to poor methodological rigour, and lack of appropriate follow-up procedures identified in the studies included in this review and the limited evidence concerning long-term outcomes and post-operative complications. We acknowledge that designing a randomised controlled trial suitable for establishing the efficacy of a surgical intervention is challenging due to inherent ethical issues surrounding experimental surgical interventions. However more high quality research is required to support the use of the flexor tenotomy procedure as a mainstream treatment option for achieving and maintaining digital ulcer healing in people with diabetes and neuropathy.

## Abbreviations

ABPI, ankle brachial pressure index; CRD, centre for reviews and dissemination; FDB, flexor digitorum brevis; FDL, flexor digitorum longus; FHB, flexor hallucis brevis; FHL, flexor hallucis longus; MINORS, methodological index for non-randomised studies; No., number; PAD, peripheral arterial disease; PRISMA, preferred reporting items for systematic reviews and meta-analyses; SIGN, Scottish intercollegiate guidelines network; UT, University of Texas

## Additional files

Additional file 1: Excluded article list. Data Table containing excluded article list. (DOCX 20 kb) Additional file 2: Final agreed MINORS Evaluation for Non-Comparative Studies. Data Table containing MINORS evaluation. (DOCX 18 kb)
