# Supplementary material for: Effectiveness of percutaneous flexor tenotomies for the management and prevention of recurrence of diabetic toe ulcers: a systematic review
Source: J Foot Ankle Res. 2016 Jul 29;9:25. doi: 10.1186/s13047-016-0159-0 (PMC4966795; doi:10.1186/s13047-016-0159-0)
Supplement: Additional file 1: — Excluded article list. Data Table containing excluded article list. (DOCX 20 kb) [file 13047_2016_159_MOESM1_ESM.docx]

Excluded Article List

|  | Paper | Source | Reason for Exclusion |
| --- | --- | --- | --- |
| 1. | Boffeli TJ, Collier RC (2015) Surgical treatment guidelines associated with intrinsic muscle spasticity (Intrinsic plus foot) in adults with cerebral palsy. *Journal of Foot and Ankle Surgery* **54**: 985-993 | Pubmed, EBSCO | **Title screen: Cerebral palsy patients** |
| 2. | Boffeli TJ, Collier RC (2014) “Minimally invasive soft tissue release of foot and ankle contracture secondary to stroke”  *Journal of Foot and Ankle Surgery* **53**: 369-75 | Pubmed, EBSCO | **Abstract review: Stroke patients** |
| 3. | Cavanagh PR, Lipsky BA, Bradbury AW, Botek G (2005) Treatment for diabetic foot ulcers. *The Lancet* **366**: 1725–35 | EBSCO | **Abstract review: General overview of treatments** |
| 4. | Dhukaram V, Hossain S, Sampath J, Barrie J (2003) Metatarsophalangeal Release and Proximal Interphalangeal Artthroplasty for Hammertoe Correction *Journal of Bone and Joint Surgery – British Volume* **85**(Suppl 2): 126 | Reference Search – (Schepers et al. 2010) 29/01/15 | **Abstract review: Arthroplasty procedure** |
| 5. | Diabetic Foot Journal (2014) Digest *The Diabetic Foot Journal* **17**: 79 | EBSCO | **Full text review: Digest of Tamir, 2014** |
| 6. | Feeney MS, Williams RL, Stephen MM (2001)  Selective lengthening of the proximal flexor tendon in the management of acquired claw toes. *Journal of Bone and Joint Surgery – British Volume* **83**: 335-8 | Reference Search – (Schepers et al. 2010) 29/01/15 | **Abstract review: Open ankle procedure** |
| 7. | Frykberg RG, Bevilacqua NJ, Habershaw G (2010) Surgical off-loading of the diabetic foot. *Journal Of The American Podiatric Medical Association.* **100**: 369-84 | Pubmed, EBSCO | **Full text review: Review of general offloading procedures** |
| 8. | Journal of Bone and Joint Surgery (2004) Orthopaedic Procedings *Jounral of Bone and Joint Surgery* **86** (Suppl 1): 1-103 | EBSCO | **Full text review: Manual search of this issue could locate nothing of relevance to the topic** |
| 9. | Kim JY, Hwang S, Lee Y (2012 ) Selective plantar fascia release for nonhealing diabetic plantar ulcerations  *Journal of Bone and Joint Surgery – American Volume* **18**: 1297-302. | Pubmed, EBSCO | **Abstract review: Plantar fascia procedure** |
| 10. | The Lancet (2000) *Lancet.* **384 (**Suppl 4) **354**: 1-66. | EBSCO | **Full text review: Manual search of this issue could locate nothing of relevance to the topic** |
| 11. | Laborde JM (2009) Letters to the editor, Lountzis et al.. Percutaneous Flexor Tenotomy – office procedure for diabetic toe ulcerations *Wounds* **19**: 64-68 | EBSCO | **Title review: editorial article** |
| 12. | Lountzis N, Parenti J, Cush G, et al (2007) Percutaneous Flexor Tenotomy -- office procedure for diabetic toe ulcerations. *Wounds: A Compendium of Clinical Research & Practice* **19**: 64-8 | EBSCO | **Full text review: description of procedure, not research study** |
| 13. | Magin MN (2014) Full weight-bearing after Lapidus arthrodesis in hallux valgus deformity using the IVP plate fixation (V-TEK(®) system). *Operative Orthopädie Und Traumatologie* **26**: 184-95. | Pubmed, EBSCO | **Abstract review: Arthrodesis Procedure** |
| 14. | Mallette LE, Patten BM, Engel WK (1975) Neuromuscular disease in secondary hyperparathyroidism *Annals of Internal Medicine* **82**: 474-483 | EBSCO | **Abstract review: Bone biopsy procedure** |
| 15. | Maffulli N, Testa V, Capasso G et al (2008) Surgery for chronic Achilles tendinopathy produces worse results in women. *Disability & Rehabilitation.* **30**: 1714-1720 | EBSCO | **Abstract review: Achilles tendon study** |
| 16. | Ozdolop S, Mathew KM, McClelland M, Ravichandran G (2006) Modified Girdlestones–Taylor procedure for claw toes in spinal cord injury. *Spinal Cord* **44**: 787-790 | EBSCO | **Abstract review: Girdlestone-taylor is a tendon transfer procedure** |
| 17. | Pearson CM, Bluestone R, Amstutz HC et al (1975) Diagnosis and treatment of Erosive Rheumatoid Arthritis and Other Forms of Joint Destruction. *Annals of Internal Medicine* **82**: 241 | EBSCO | **Abstract review: Rheumatoid Patients** |
| 18. | Roukis TS, Schade VL (2009) Percutaneous Flexor Tenotomy for treatment of neuropathic toe ulceration secondary to toe contracture in persons with diabetes: a systematic review. *The Journal of Foot and Ankle Surgery* **48**: 684–689 | Pubmed, EBSCO | **Abstract review: Systematic Review** |
| 19. | Rubin G, Wolovelsky A, Rinott M, Rozen N (2010) Chronic granulomatous tenosynovitis treated with ulnar superficialis slip resection. *Orthopedics.* **33** | Pubmed, EBSCO | **Abstract review: Finger procedure** |
| 20. | Schepers T, Berendsen HA, Hok Oei I, Koning J (2010) Functional outcome and patient satisfaction after Flexor Tenotomy for plantar ulcers of the toes. *The Journal of Foot and Ankle Surgery* **49**: 119–122 | Pubmed, EBSCO | **Full text review: Diabetic results not separated from rheumatoid results** |
| 21. | Stauff MP, Kilgore WB, Joyner PW, Juliano PJ (2011) Functional outcome after percutaneous tendo-Achilles lengthening. *Foot And Ankle Surgery.* **17**: 29-32 | Pubmed, EBSCO | **Abstract review: Tendo-Achilles Procedure** |
| 22. | Tamir E, McLaren A, Gadgil A, Daniels TR (2008) Outpatient percutaneous flexor tenotomies for management of diabetic claw toe deformities with ulcers: a preliminary report. *Canadian Journal of Surgery* **51**: 41–44 | Pubmed, EBSCO | **Full text review: Adjunct procedure - osteoclasis** |
| 23. | Terjesen T, Lie G, Hyldmo Å, Knaus A (2005) Adductor tenotomy in spastic cerebral palsy. *Acta Orthopaedica*. **76**: 128-137 | EBSCO | **Abstract review: Cerebral palsy patient** |
| 24. | Vaseenon T, Phisitkul P (2010) A novel tendinous interconnection release technique for claw-toe deformity *The Iowa Orthopaedic Journal.* **30**: 157 60. | EBSCO | **Full text review: Tendinous interconnection release, not tenotomy** |
