# Supplementary material for: Effectiveness of percutaneous flexor tenotomies for the management and prevention of recurrence of diabetic toe ulcers: a systematic review
Source: J Foot Ankle Res. 2016 Jul 29;9:25. doi: 10.1186/s13047-016-0159-0 (PMC4966795; doi:10.1186/s13047-016-0159-0)
Supplement: Additional file 2: — Final agreed MINORS Evaluation for Non-Comparative Studies. Data Table containing MINORS evaluation. (DOCX 18 kb) [file 13047_2016_159_MOESM2_ESM.docx]

MINORS Evaluation for Non-Comparative Studies

| **Kearney, et al (2010)** | | |
| --- | --- | --- |
| 1. Clearly stated aim | “To evaluate the effectiveness and safety of percutaneous tenotomy of the flexor digitorum longus to heal neuropathic ulcers on the tip of the toe” | 2 |
| 2. Inclusion of consecutive patients | “48 consecutive patients”. Inclusion and exclusion criteria listed for procedure. Not stated if any patients were excluded. | 1 |
| 3. Prospective collection of data | Retrospective | 0 |
| 4. Endpoints appropriate to the aim of the study | Endpoints: Healing achieved, average healing time, recurrent ulcer at same site, time to recurrence, infection rate, amputations. Appropriate when considering efficacy and safety. | 2 |
| 5. Unbiased assessment of the study endpoint | Evaluation appears to be done by in-house personnel. Appears to be ascertained from medical records. No information on blinding. | 0 |
| 6. Follow-up period appropriate to study aim | “Mean Follow up was 28 Months” – More detail needed, no information on shortest follow up or on follow up method. | 1 |
| 7. Loss to follow up <5% | Not reported | 0 |
| 8. Prospective calculation of study size | No | 0 |
| **SCORE** | | **6** |
| *Key: 0 – Not reported, 1 – Reported but inadequate, 2 – Reported and adequate. Optimal Score – 16* | | |

| **Laborde (2007)** | | |
| --- | --- | --- |
| 1. Clearly stated aim | This study “evaluates the results of treating plantar neuropathic toe ulcers with percutaneous Flexor Tenotomy” – Vague. | 1 |
| 2. Inclusion of consecutive patients | Some information on patient selection criteria, but not explicit inclusion and exclusion. Not reported if patients were consecutive. | 0 |
| 3. Prospective collection of data | Retrospective | 0 |
| 4. Endpoints appropriate to the aim of the study | Healing, healing time, recurrence, complications. Appropriate when considering evaluation of procedure results. | 2 |
| 5. Unbiased assessment of the study endpoint | No blinding. Outcomes self reported by telephone by some patients. | 0 |
| 6. Follow-up period appropriate to study aim | Average 36 Months (range 20-65 months). Comprehensive recording of patient characteristics. 6 Patients had phone follow up. | 2 |
| 7. Loss to follow up <5% | No patients lost to follow up | 2 |
| 8. Prospective calculation of study size | No | 0 |
| **SCORE** | | **7** |
| *Key: 0 – Not reported, 1 – Reported but inadequate, 2 – Reported and adequate. Optimal Score – 16* | | |

| **Rasmussen et al (2013)** | | |
| --- | --- | --- |
| 1. Clearly stated aim | “To examine the effectiveness of Flexor Tenotomy in a modified technique to prevent and heal neuropathic and neuroischemic pressure ulcers on the tip of the toe in claw or hammer toe deformities in people with diabetes” | 2 |
| 2. Inclusion of consecutive patients | Consecutive 4-year series. Comprehensive patient identification method described. | 2 |
| 3. Prospective collection of data | Retrospective | 0 |
| 4. Endpoints appropriate to the aim of the study | Healing rate, healing time, recurrence rate, infection rate, amputations, ulcer incidence, complications. Appropriate when considering effectiveness of procedure on healing rates. Prevention harder to establish. | 2 |
| 5. Unbiased assessment of the Study endpoint | No discussion of who conducted endpoint evaluation | 0 |
| 6. Follow-up period appropriate to study aim | Follow up: Median 31 months, range 2-48 months. Two months is inadequate. No explanation given for the follow up period. | 1 |
| 7. Loss to follow up <5% | No discussion of losses. The fact at least one patient was only followed up by two months may indicate this was a loss. | 0 |
| 8. Prospective calculation of study size | No | 0 |
| **SCORE** | | **7** |
| *Key: 0 – Not reported, 1 – Reported but inadequate, 2 – Reported and adequate. Optimal Score – 16* | | |

| **Tamir et al (2014)** | | |
| --- | --- | --- |
| 1. Clearly stated aim | "The purpose of this study was to report on performing percutaneous flexor tenotomies for treating neuropathic toe ulcers" | 2 |
| 2. Inclusion of consecutive patients | Patient identification method appears to be consecutive but not explicitly stated. | 0 |
| 3. Prospective collection of data | Retrospective | 0 |
| 4. Endpoints appropriate to the aim of the study | Healing rate, complications, recurrence rate - appropriate | 2 |
| 5. Unbiased assessment of the Study endpoint | Chart review by staff at surgical unit – not unbiased. | 0 |
| 6. Follow-up period appropriate to study aim | Median – 22 months, minimum 5 months, interquartile range 16-29. | 1 |
| 7. Loss to follow up <5% | Not reported | 0 |
| 8. Prospective calculation of study size | No | 0 |
| **SCORE** | | **5** |
| *Key: 0 – Not reported, 1 – Reported but inadequate, 2 – Reported and adequate. Optimal Score – 16* | | |

| **Van Netten et al (2013)** | | |
| --- | --- | --- |
| 1. Clearly stated aim | "The aim of this study was to retrospectively investigate all consecutive flexor tenotomies in people with neuropathic diabetic foot ulcers on the distal end over the toe, to report healing and time to heal, and to investigate the influence of preoperative treatment, ulcer duration before Flexor Tenotomy, ulcer location and infection on healing and time to heal". Comprehensive. | 2 |
| 2. Inclusion of consecutive patients | Consecutive. Clearly outlined. | 2 |
| 3. Prospective collection of data | Retrospective | 0 |
| 4. Endpoints appropriate to the aim of the study | Healing rate, healing time, complications, new ulcer incidence. Appropriate when considering study aims. | 2 |
| 5. Unbiased assessment of the Study endpoint | Chart review, no details of blinding | 0 |
| 6. Follow-up period appropriate to study aim | Mean: 23 ± 11 months (range 11-60months). Appropriate | 2 |
| 7. Loss to follow up <5% | Not reported | 0 |
| 8. Prospective calculation of study size | No | 0 |
| **SCORE** | | **8** |
| *Key: 0 – Not reported, 1 – Reported but inadequate, 2 – Reported and adequate. Optimal Score – 16* | | |
